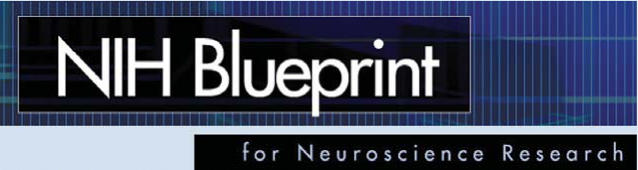# The NIH Blueprint for Neuroscience Research

**Published:** 2006-03

Disorders of the nervous system affect one in three Americans and present an extraordinary challenge for health science research. As part of its broad mission to understand the interplay between environmental factors and genetic susceptibility in human health and disease, the NIEHS has an active extramural research program in neuroscience, particularly in the areas of neurodevelopment and neurodegeneration. The NIEHS recently joined the Neuroscience Blueprint, a formal collaboration among 15 NIH institutes that support research on the nervous system. Scientific programs implemented under the Blueprint are intended to accomplish goals that strengthen the neuroscience enterprise and make it more effective and that also affect many or all of the individual disciplines within neuroscience. These goals include pooling of resources and expertise, establishing economies of scale, addressing large and complex challenges within neuroscience, and developing tools and infrastructure to serve the entire neuroscience community.

During its first full year of operation, the Neuroscience Blueprint has acted to expand a number of existing resources and has established an infrastructure for the efficient development and implementation of new initiatives. One of the initial Blueprint activities was the expansion of the Microarray Consortium. Four microarray centers are now available to serve the expression profiling and single nucleotide polymorphism (SNP) genotyping needs of neuroscience investigators funded by any of the Blueprint institutes. The consortium provides advice on experimental design, experimental services, data analysis, manuscript preparation support, and training. Extensive expertise, laser-capture microdissection, and multiple microarray platforms are available. NIEHS-funded investigators with an active grant in neuroscience are eligible to access the consortium to perform neuroscience experiments, and, because NIH pays most consortium costs, user fees are very reasonable.

A number of Neuroscience Blueprint initiatives are being implemented in 2006. These include training initiatives in neuroimaging and computational science, the development and dissemination of knockout mouse lines, the establishment of core facilities for neuroscience research, and the development of a standard data collection instrument for neuroepidemiologic research. Planning for Fiscal Year 2007, new Blueprint initiatives will focus on tools and resources for neurodegeneration. Future initiatives for subsequent fiscal years will focus on development and plasticity.

Additional information about the Neuroscience Blueprint can be found at **http://neuroscienceblueprint.nih.gov/**

## Contact

**Cindy Lawler, PhD** | (919) 316-4671, lawler@niehs.nih.gov

## Figures and Tables

**Figure:**